# Ketogenic diet in a patient with congenital hyperinsulinism: a novel approach to prevent brain damage

**DOI:** 10.1186/s13023-015-0342-6

**Published:** 2015-09-24

**Authors:** Arianna Maiorana, Lucilla Manganozzi, Fabrizio Barbetti, Silvia Bernabei, Giorgia Gallo, Raffaella Cusmai, Stefania Caviglia, Carlo Dionisi-Vici

**Affiliations:** Metabolic Unit, Department of Pediatric Medicine, Bambino Gesù Children’s Hospital, piazza S. Onofrio 4, 00165 Rome, Italy; Department of Experimental Medicine, University of Tor Vergata and Bambino Gesù Children’s Hospital, Rome, Italy; Clinical Nutrition, Gastroenterology Department, Bambino Gesù Children’s Hospital, Rome, Italy; Neurology, Neuroscience Department, Bambino Gesù Children’s Hospital, Rome, Italy; Psychology Unit, Neuroscience Department, Bambino Gesù Children’s Hospital, Rome, Italy

**Keywords:** Congenital hyperinsulinism, Ketogenic diet, Hypoglycemia, Epilepsy, Neurodevelopment

## Abstract

**Background:**

Congenital hyperinsulinism (CHI) is the most frequent cause of hypoglycemia in children. In addition to increased peripheral glucose utilization, dysregulated insulin secretion induces profound hypoglycemia and neuroglycopenia by inhibiting glycogenolysis, gluconeogenesis and lipolysis. This results in the shortage of all cerebral energy substrates (glucose, lactate and ketones), and can lead to severe neurological sequelae. Patients with CHI unresponsive to medical treatment can be subjected to near-total pancreatectomy with increased risk of secondary diabetes. Ketogenic diet (KD), by reproducing a fasting-like condition in which body fuel mainly derives from beta-oxidation, is intended to provide alternative cerebral substrates such ketone bodies. We took advantage of known protective effect of KD on neuronal damage associated with GLUT1 deficiency, a disorder of impaired glucose transport across the blood-brain barrier, and administered KD in a patient with drug-unresponsive CHI, with the aim of providing to neurons an energy source alternative to glucose.

**Methods:**

A child with drug-resistant, long-standing CHI caused by a spontaneous *GCK* activating mutation (p.Val455Met) suffered from epilepsy and showed neurodevelopmental abnormalities. After attempting various therapeutic regimes without success, near-total pancreatectomy was suggested to parents, who asked for other options. Therefore, we proposed KD in combination with insulin-suppressing drugs.

**Results:**

We administered KD for 2 years. Soon after the first six months, the patient was free of epileptic crises, presented normalization of EEG, and showed a marked recover in psychological development and quality of life.

**Conclusions:**

KD could represent an effective treatment to support brain function in selected cases of CHI.

## Background

Congenital hyperinsulinism (CHI) is the most frequent cause of hypoglycemia in children [1, 2]. Mutations that affect insulin secretion regulation by the three main classes of energy substrates, i.e. glucose, aminoacids, and free fatty acids (FFA) [2, 3], can cause CHI that requires rapid diagnosis and treatment to limit/avoid neuronal damage [4] and the irreversible neurological sequelae consequent to prolonged, severe hypoglycemia. Neurons in the superficial layers of cerebral cortex and hippocampus are those preferentially affected by lack of glucose, followed by neurons in basal ganglia and thalamus [5]. However, mild, recurrent hypoglycemia can cause hippocampal synaptic dysfunction even in absence of neuronal damage [6, 7]. These experimental findings explain why memory, learning, intelligence and attention are the cognitive domains most vulnerable to hypoglycemia in children with type 1 diabetes [8, 9]. Although glucose is the main energy source for neurons, human brain can also utilize ketone bodies from FFA, lactate, pyruvate, glycerol and some aminoacids, as alternative substrate [10]. The protective effect of ketone bodies on hypoglycemia-induced neuronal damage has been demonstrated in animal studies [11, 12] and also in patients with type 1 diabetes, in whom the ingestion of medium-chain triglycerides prevented the cognitive deficit induced by hypoglycemia by elevating blood levels of 3-hydroxybutyrate [13]. Ketogenic diet (KD), which provides FFA as alternative fuel to carbohydrates for neuronal energy metabolism, has therefore a strong potential neuroprotective effect. The main indication of KD in children is the treatment of refractory epilepsy, but it is also the causal therapy of GLUT1 deficiency, a metabolic disorder characterized by epilepsy, developmental delay and movement disorders [14, 15]. In GLUT1 deficiency, neuroglycopenia that ensues as consequence of the impaired glucose transport across the blood-brain barrier [14, 15] is effectively improved by KD that provides ketone bodies as alternative energy source for the brain. In CHI, excessive insulin secretion not only induces severe neuroglycopenia, but also halts, by inhibiting gluconeogenesis, glycogenolysis and lipolysis, the use of other metabolic pathways that provide energetic substrates to the neurons. Other inherited metabolic diseases, such as mitochondrial fatty oxidation defects, share the same neurological risk of hypoglycemia because of lack of ketones [16]. Therefore, developing brain of patients with CHI is more vulnerable than other forms of hypoglycemia. Based on the similarities of brain metabolism perturbation shared by GLUT1 deficiency and CHI, we attempted to tackle neuroglycopenic symptoms and outcome by administering KD in a patient with severe, drug-resistant form of CHI.

## Methods

We report a case of severe persistent hyperinsulinemic hypoglycemia due to a “de novo” mutation in *GCK*. The girl presented with hypoglycemic seizures since the first years of life. Family history was positive for type 2 diabetes. From the age of 2 years, she was admitted to the hospital for recurrent episodes of severe hypoglycemia (up to 1.5 mmol/L, normal value 3.9–5.5 mmol/L [17]) associated to cold sweating, seizures and hypotonia. At the age of 3 years laboratory tests were performed and showed hypoglycemia with hyperinsulinemia (blood glucose 1.95–2.3 mmol/L; plasma insulin 5.5–10.2 μUI/ml). EEG showed abnormal waves in the left hemisphere and epileptogenic abnormalities, and treatment with valproate was started. Genetic investigation for *ABCC8* and *KNCJ11* performed elsewhere was negative. At 9 years of age the patient was referred to our hospital, and the heterozygous p.Val455Met mutation was found at *GCK* gene sequencing [18]; parents showed wild type *GCK* sequence. At admission, the child was receiving a combination of high-dose diazoxide (12 mg/kg/day) and octreotide (15 μg/kg/day), with no apparent clinical response. Previously, a trial with nifedipine (up to 0.75 mg/kg/day) had been attempted without benefit. We gradually increased octreotide to 50 μg/kg/day in combination with diazoxide (10 mg/kg/day) but observed persistence of hypoglycemia and epilepsy. A further association with slow-release carbohydrate to drugs did not elicit any clinical improvement, and the patient continued to present hypoglycemic episodes (0.5–1.6 mmol/L), seizures and absence epilepsy regardless of glycemic values, that required frequent hospitalizations. Despite a mild improvement of neurologic symptoms after switch from valproate therapy to ethosuccimide, absence epilepsy and evident EEG abnormalities persisted, even in the intercritical phases; her intellectual function was borderline. Furthermore, at the age of 10 years the patient quickly gained 11.5 kg within 12 months, becoming mildly obese (BMI z-score: >97^th^ centile). Overall, her quality of life was very poor. The lack of response to drug therapy with risk of permanent and severe brain sequelae made us to consider a near-total pancreatectomy, that was discussed with parents with the warning of no guarantee to achieve normoglycemia and of the increased hazard of secondary diabetes. At parents’ request to avoid surgery, we then proposed a trial with KD, explaining that it was aimed to prevent neuroglycopenic epilepsy and to improve neurological status by providing ketone bodies as an alternative energy source for neurons, as seen in GLUT1 deficiency. Consent was obtained from patient and parents after full explanation of the objective. With the new dietary regimen we provided 85 % of energy from lipids, 8 % from proteins, and 7 % from glucose of a normocaloric diet divided in 5 meals. Determination of blood ketones (Nova StatStrips® Glucose Ketone Meter, Nova Medical, Menarini Diagnostics, Florence, Italy) in the morning (after overnight fast of 10–12 h) and in the evening (after dinner) was utilized as guidance to establish the ratio of lipids: proteins plus carbohydrates. The ratio was progressively increased every three months from 1.5:1 to 3:1 during the first year of KD in order to obtain a blood ketone concentration close to 4 mmol/L [19]. First assessment of the KD outcome was performed as inpatient; auxological evaluation, hepatic and renal function, plasma glucose, insulin, lactate, FFA, blood ketones and lipid profile, along with renal tubular function and abdomen ultrasound were assessed every 6 months. Follow-up included 96 to 120-h long continue glucose monitoring (CGM- ipro2, Medtronic; data analysis by MiniMed software, Medtronic, MiniMed, Northridge, CA, USA®) [20] with EEG performed simultaneously. Weschsler Intelligence scale for Children (WISC III: third edition) and Vineland Adaptive Behavior Scale were serially administered to evaluate cognitive and adaptive abilities.

## Results and discussion

**Fig. 1:**
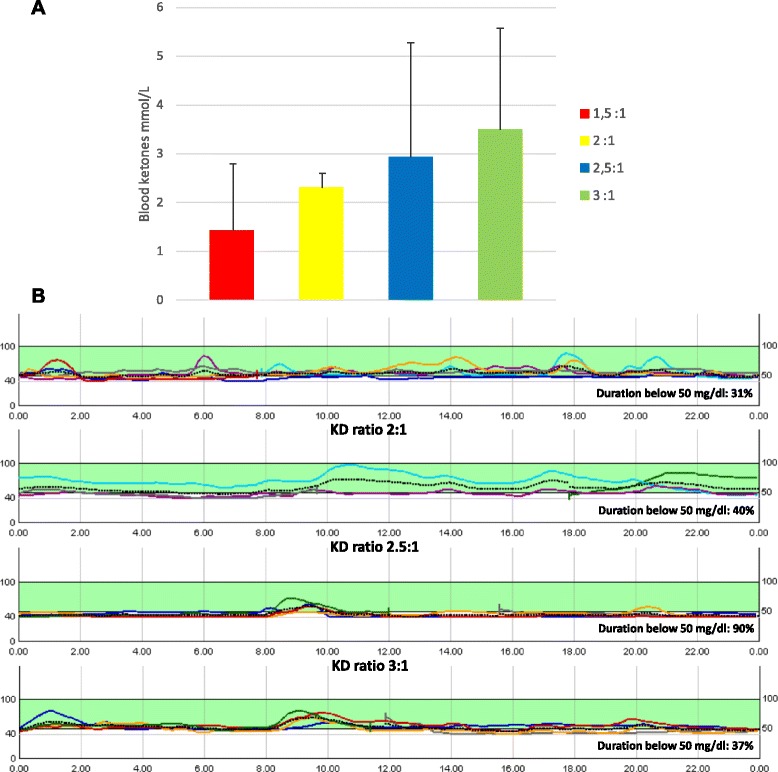
Blood ketones and glucose levels during KD. Ketones progressively raised up to 2–5 mmol/L while increasing the KD ratio from 1.5:1 to 3:1 (Panel **a**). 120h-continuous glucose monitoring on KD at 2 weeks, 3 months, 6 months, 12 months showing persistence of hypoglycemia (mean 52 mg/dl, 2.8 mmol/L, range 40–84 mg/dl, 2.2–4.6 mmol/L) (Panel **b**)

**Fig. 2:**
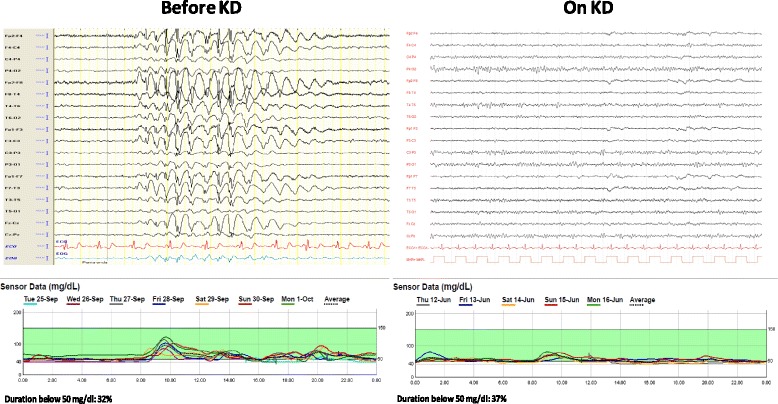
Comparison between cerebral activity and glycemia before and after 1 year of KD. Disappearance of absence epilepsy or electrical signs despite hypoglycemia. Before KD, long monitoring EEG showed generalized spike and waves discharges with loss of contact during hypoglycemia. On KD, long monitoring EEG appeared normal, with absence of ictal EEG and epileptic manifestations even during hypoglycemia

**Fig. 3:**
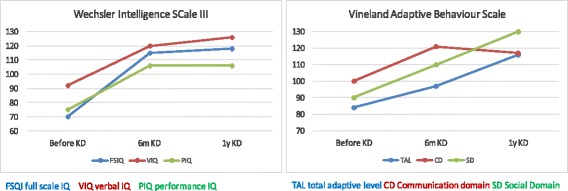
Cognitive an adaptive skills before and after 1 year of KD. Normalization of cognitive, social and verbal capacities on KD. Weschsler Intellingence Scale for Children (WISCIII): normal >80, borderline 70–80; Vineland Adaptive Behavior Scale (VABS): moderately high 116–130, normal 85–115, moderately low 70–84

While on KD the patient continued diazoxide and octreotide treatment (10 mg/kg/day and 35 μg/kg/day, respectively). Six months after KD was started, maintenance of blood ketones between 2–5 mmol/L (Fig. 1, panel a) fully resolved neuroglycopenic signs with parallel disappearance of both epileptic crisis and absence epilepsy, despite blood glucose levels permanently below 5.5 mmol/L even after meal, and close to 2.2–2.7 mmol/L most of time (Fig. 1, panel b). EEG improved and became normal within the first year on KD, showing no alteration even during episodes of hypoglycemia (Fig. 2). During the first 6 months of KD the patient lost 9 kg and her BMI normalized. Psychological evaluation revealed a strengthening of social, cognitive and verbal capacities (Fig. 3). The child and her family reported an improvement of physical and psychosocial well-being, reduction of fear of hypoglycemic symptoms and awareness of a lower risk of neurological injury, with an overall amelioration of the quality of life related to the management of disease. Diazoxide was discontinued, and currently the patient is given octreotide, reduced to 25 μg/kg/day, without any neuroglycopenic symptoms. KD was well tolerated over a period of 24 months, with no side-effects and no changes in laboratory tests.

GCK activation caused by gain-of-function mutations determines an increased affinity for glucose and lowers the glucose thresholds for insulin release in the pancreatic beta cell, giving rise to CHI [18, 20–23]. Most patients bearing a *GCK* mutation have relatively mild disease, easily managed with diazoxide, but a few cases with a severe phenotype, who were subjected to near-total pancreatectomy to avoid neurological sequelae, have been described [24, 25]. Although V455M mutation has been previously associated with a mild phenotype [18], our patient displayed a drug-resistant form, with a steep increase of BMI in peripubertal age and a clinical course resembling that described in a patient with the *GCK*/A456V mutation [26]. Prompted by patient’s long-standing symptoms, drug resistance and the preference of parents to avoid demolitive surgery, we opted for a KD trial to provide an energy source to central nervous system (CNS) alternative to glucose, on the bases of its proven efficacy for the treatment of GLUT1 deficiency [14]. We thus challenged the central tenet of treatment of CHI patients, which is to maintain normoglycemia to avoid irreversible brain damage [4]. As a matter of fact, neurological sequelae are more prevalent in CHI than in other forms of hypoglycemia because of the inhibition of lipolysis and ketogenesis by inappropriately high insulin levels [16]. Ketogenic diet, by restricting the amount of carbohydrates along with a significant increase of fat intake, reproduces a fasting-like condition in which metabolism is shifted from glycolysis to beta-oxidation of FFA and ketone bodies formation for ATP synthesis [27]. In our patient we observed that after the beginning of KD neurological symptoms, psychological development and epilepsy manifestations all improved, despite the persistence of hyperinsulinemic hypoglycemia. The neuroprotective effect of ketone bodies has been associated with the activation of a several endogenous metabolic and genetic programs that stabilize and/or enhance cellular metabolism [27], increasing cerebral ATP and reducing neuronal excitability. In addition, KD decreases mTOR activity in neuronal cells, thus conferring anticonvulsant effect [28]. Of note, KD reverted EEG pathologic patterns in our patient even during hypoglycemia (Fig. 2). This result highlights the protective effect of KD on CNS despite persistence of neuroglycopenia. Cognitive and adaptive development also improved after the first six months of KD, further confirming its positive effect. Bearing in mind that ketone bodies have additional neuroprotective effects acting as ROS scavengers [11], and by inducing PPARα and UCP2 expression [29], and inhibiting glutamate uptake into synaptic vesicles [30], we hypothesize that KD might have prolonged positive effects on brain function.

CNS protective action of 3-hydroxybutyrate has been successfully exploited to treat hypoglycemic coma in rats [31] and multiple acyl-CoA dehydrogenase deficiency in humans [32]. Consequently, we suggest that the utilization of 3-hydroxybutyrate may be a safer option and an area of investigation in specific patients, such neonates with CHI, in whom administration of KD might be hazardous.

## Conclusions

The clinical and neurological improvement observed in our patient with CHI suggests that KD could have a neuroprotective effect despite persistence of neuroglycopenia. Additional studies are needed to confirm the efficacy of this novel therapeutic approach in selected CHI cases to support brain function by providing an alternative energy source to CNS.

## Abbreviations

CHI: Congenital hyperinsulinism
KD: Ketogenic diet
GCK: Glucokinase
GLUT1: Glucose transporter 1
EEG: Electroencephalography
FFA: Free fatty acids
ROS: Radical oxygen species
CNS: Central nervous system
